# Comparative study of the anti-tumour effects of the imipridone, ONC201 and its fluorinated analogues on pancreatic cancer cell line

**DOI:** 10.1038/s41598-025-00070-x

**Published:** 2025-05-07

**Authors:** Zsófia Szász, Angéla Takács, Márton Kalabay, Péter Bárány, Tamás Czuczi, Antal Csámpai, Eszter Lajkó, László Kőhidai

**Affiliations:** 1 Department of Genetics, Cell- and Immunobiology, Semmelweis University, Budapest, 1089 Hungary; 2 Department of Organic Chemistry, Institute of Chemistry, Eötvös Loránd University, Budapest, 1117 Hungary

**Keywords:** Pancreas adenocarcinoma, Imipridone, Apoptosis, ONC201, Drug development, Tumour selectivity, Drug development, Targeted therapies, Pancreatic cancer

## Abstract

Pancreatic ductal adenocarcinoma has a high mortality rate, with a 5-year survival rate of ~ 12%. Therefore, developing new targeted therapies is urgently needed. ONC-201, a promising candidate, is currently undergoing clinical trials. The main objective of the present work is to investigate the anti-tumour activity of ONC-201 and its two fluorinated analogues (TBP-134, TBP-135). The viability of two pancreatic adenocarcinoma cell lines (PANC-1, MIA PaCa-2) and three other tumour cell lines (A2058, EBC-1, COLO-205) was assessed after 72-hour treatment with drugs at 0.5, 10, and 25 µM. Significant antiproliferative effects were observed, with 0.5 µM TBP-134 achieving the highest potency, reducing cell viability to approximately 50%. None of the molecules exhibited significant cytotoxicity toward normal human dermal fibroblast cells or cardiomyocytes, indicating a selective anti-tumour profile. The analogues showed more effective results than ONC201 on PANC-1 cells (IC_50_: 0.35 and 1.8 µM vs. IC_50_: 6.1 µM, respectively). All analogues induced G2/M phase arrest followed by apoptosis in PANC-1 cells. The site of the fluorination influenced the mechanism of apoptotic action of these compounds. Overall, TBP-134 showed superior efficacy, making it a promising candidate for structural optimization within the imipridone family to develop more effective, selective treatments for pancreatic tumours.

**Supplementary Information:**

The online version contains supplementary material available at 10.1038/s41598-025-00070-x.

## Introduction

Pancreatic adenocarcinoma remains one of the tumours with the worst prognosis, with the 5-year survival rate rising to around 11% in recent years^1,2^. More than half of the cases are diagnosed at stage IV, which means metastases have already been developed^3^. The most current therapeutic approach is the surgical removal of the tumour combined with adjuvant chemotherapy^4^. This poor prognosis highlights the need to change late diagnosis and treatment protocols.

Small-molecule drugs intended for targeted tumour therapy are among the new anti-cancer agents. Due to their size, these molecules have a high membrane penetration, which is much more favourable than biologics. Their bioavailability is more beneficial than antibodies’, resulting in faster absorption and elimination. In this regard, ONC201 or TRAIL Inducing Compound 10 (TIC-10), which is a small molecular weight (M = 386.5 g/mol) imidazolinopyrimidinone comprising an angular tricyclic central heterocyclic skeleton has beneficial features. This compound is the first-in-class representative of the emerging imipridone family of anticancer agents^5^. Due to its water-solubility and lipophilicity (logP = 2.3), this anticancer agent is suitable for oral administration, as is demonstrated by currently undergoing clinical trials involving patients with recurrent glioblastoma^6,7^. Because of these physicochemical properties, ONC201 is readily distributed throughout the body, exerting its effects across the blood-brain barrier. Consequently, this small molecule anticancer agent might have a wide range of applications in treating various tumours.

The mechanism of action of ONC201 is still under active investigation. According to the literature, this molecule acts on tumour cells through two main pathways^8,9^. The drug induces the intrinsic pathway of apoptosis in a p53-dependent manner and increases the activity of the initiator caspase-9. This mode of action is less effective in most tumours because a very high percentage of the tumour cells have mutated p53 protein, so the intrinsic pathway is blocked, and apoptotic cell death cannot occur. Furthermore, according to the literature, the direct target molecule of ONC201 is the caseinolytic protease P (ClpP), upon which ONC201 acts as an allosteric agonist^10^. ClpP is a serine protease located in the mitochondrial matrix. ONC201 can form a complex with the ClpP, which hyperactivates it and leads to reduced mitochondrial respiratory function and induction of cell death via the intrinsic pathway^11^.

The other way to exert its anti-tumour effect is by simultaneously inhibiting the protein B kinase (Akt) and extracellular signal-regulated kinase (ERK) pathways^5,12^. Through this pathway, ONC201 can also increase the transcription of the TNF-related apoptosis-inducing ligand (TRAIL) protein. The TRAIL protein then binds to its receptor DR4/5 (TRAIL-R1/2) in the cell membrane, where the receptor-ligand complex induces apoptosis via the TNF signalling pathway (extrinsic apoptotic pathway)^13^. TRAIL belongs to the TNF superfamily, which has two forms: soluble and membrane-bound^14^. It is physiologically expressed by the cells of the innate immune system and is necessary for maintaining immune homeostasis. Its receptors are the death receptors TRAIL-R1/DR4 and TRAIL-R2/DR5. The receptor-ligand complex leads to the death of abnormal cells in a p53-independent manner by triggering pro-apoptotic processes. By exploiting this pathway, this protein and its receptor have become the optimal target molecule for novel anti-tumour agents^15^.

Literature data suggests that the halogenation of molecules, including the production of fluorinated derivatives, is becoming increasingly common in drug development to achieve beneficial effects. Fluorination of a parent molecule can affect both the pharmacodynamics and pharmacokinetics^16^. It was also reported that there is a further increase in p53 protein activity compared to non-halogenated anti-tumour drugs^17^. This also contributes to a shift in the apoptotic effect of the molecules towards the intrinsic pathway.

In this study, we aimed to characterise the anti-tumour effect of two fluorinated derivatives of ONC201, the para- (TBP-134) and meta- (TBP-135) fluorinated structural isomers on the pancreas ductal adenocarcinoma cell lines in comparison with other cancer cell lines (e.g. melanoma) with high mortality rate. The main focus was to detect the induction of the TRAIL protein and to understand the molecular background of the induced apoptosis. Furthermore, we wanted to find out whether the halogenation site caused any difference in the effects on cell viability and played any role in the mechanism of action of the derivatives in vitro.

## Results

### The fluorinated analogues exhibit a better IC_50_ on PANC-1 cells and do not affect the cell viability of NHDF cells. Neither molecule has a direct cytotoxic effect on PANC-1 cells

To determine the effects of the tested molecules on the cell viability of two pancreas adenocarcinoma cell lines, PANC-1 and MIA PaCa-2, and three other types of tumour cell lines A2058 (metastatic melanoma), EBC-1 (lung squamous cell carcinoma) and COLO-205 (colorectal adenocarcinoma) were used. The viability measurement of the cells was carried out after 72-hour treatment with 0.5, 10 and 25 µM. All three molecules could reduce the cell viability of PANC-1 cells under 20% after treatment at 10 µM. Moreover, TBP-134 at 0.5 µM had a significant cell viability-reducing effect, and the viability of PANC-1 cells dropped to 13.4%. Meanwhile, on MIA PaCa-2 cells, the compounds demonstrated antiproliferative activity, with TBP-134 showing the best effect at 0.5 µM, with a reduced viability of 48.9% (Table 1, Supplementary Fig. S1). On the other three cell lines at 0.5 µM, only TBP-134 could exert its anti-tumour effect by reducing the viability to around 50%. The viability of the cells was the following: A2058–51.4%, EBC-1–52.2% and COLO-205–45.5%. The IC_50_ values of the compounds on all cell lines are collected in a table and found in the Supplementary Material (Supplementary Table S1).

Healthy human dermal fibroblast (NHDF) cells were used to determine the effects of the tested compounds on healthy cells. The experimental results showed that the molecules reduced the viability of NHDF cells, with TBP-134 demonstrating an antiproliferative effect on these cells. However, at the lowest concentration, the viability was 61.5%, while the other two molecules did not lower the cell viability at the same concentration.

To assess the cardiotoxic effect of the drugs, the viability of HL-1 cells was measured after a 72-hour treatment. Even with the highest concentration (25 µM), the viability of the HL-1 cells remained above 80%.

**Table 1:** The viability results of ONC201 and its analogues on different cell lines after 72-hour treatment at 0.5, 10 and 25 μM.

Cell lines	Viability (%) after 72-h treatment								
	0.5 μM			10 μM			25 μM		
	ONC201	TBP-134	TBP-135	ONC201	TBP-134	TBP-135	ONC201	TBP-134	TBP-135
PANC-1	105.0 ± 5.2	13.2 ± 1.4^z^	105.9 ± 2.4	16.0 ± 2.4^z^	7.4 ± 0.3^z^	8.8 ± 0.1^z^	10.2 ± 2.2^z^	6.1 ± 0.9^z^	9.2 ± 4.6^z^
MIA PaCa-2	77.6 ± 2.3^z^	48.9 ± 2.9^z^	63.5 ± 2.6^z^	44.6 ± 2.0 ^z^	65.0 ± 4.9^z^	62.7 ± 7.8^z^	55.0 ± 1.6^z^	59.0 ± 5.5^z^	65.5 ± 7.9^z^
A2058	101.0 ± 1.7	51.4 ± 2.4^z^	74.9 ± 1.1^z^	73.7 ± 4.5^x^	47.7 ± 3.5^z^	47.0 ± 4.7^z^	62.1 ± 8.7^x^	44.7 ± 2.8^z^	46.8 ± 2.9^z^
EBC-1	101.1 ± 1.6	52.3 ± 1.5^z^	66.6 ± 4.1^y^	47.1 ± 2.2^z^	51.7 ± 0.7^z^	71.8 ± 31.1^x^	45.5 ± 2.0^z^	50.5 ± 2.0^z^	48.0 ± 4.0^z^
COLO-205	97.0 ± 2.5	45.5 ± 1.3^z^	74.7 ± 0.6^y^	69.7 ± 2.4^z^	48.8 ± 1.6^z^	52.6 ± 4.1^z^	71.0 ± 0.7^z^	54.3 ± 0.6^z^	54.2 ± 3.2^z^
NHDF	114.7 ± 1.4*^,y^	61.5 ± 0.5^z^	111.4 ± 2.9*	88.3 ± 7.4*^,x^	46.2 ± 3.4^z^	65.9 ± 1.8*^,z^	74.9 ± 8.6^z^	48.3 ± 1.2^z^	71.4 ± 12.3^z^
HL-1	99.4 ± 1.8	89.9 ± 5.2^y^	98.8 ± 1.5	89.2 ± 6.3^y^	88.4 ± 5.0^y^	86.8 ± 3.2^z^	89.0 ± 5.6^y^	82.0 ± 2.3^z^	88.3 ± 2.8^y^

For the results marked with an asterisk (*), treatments were conducted at either 0.37 µM or 8.6 µM concentrations. The data are presented as mean values ± standard deviation (SD) (*n* = 3). One-way ANOVA test followed by Fisher’s LSD *post hoc* test was used and the levels of significance are shown as x - *p* < 0.05, y – *p* < 0.01, z – *p* < 0.001.

**Fig. 1:**
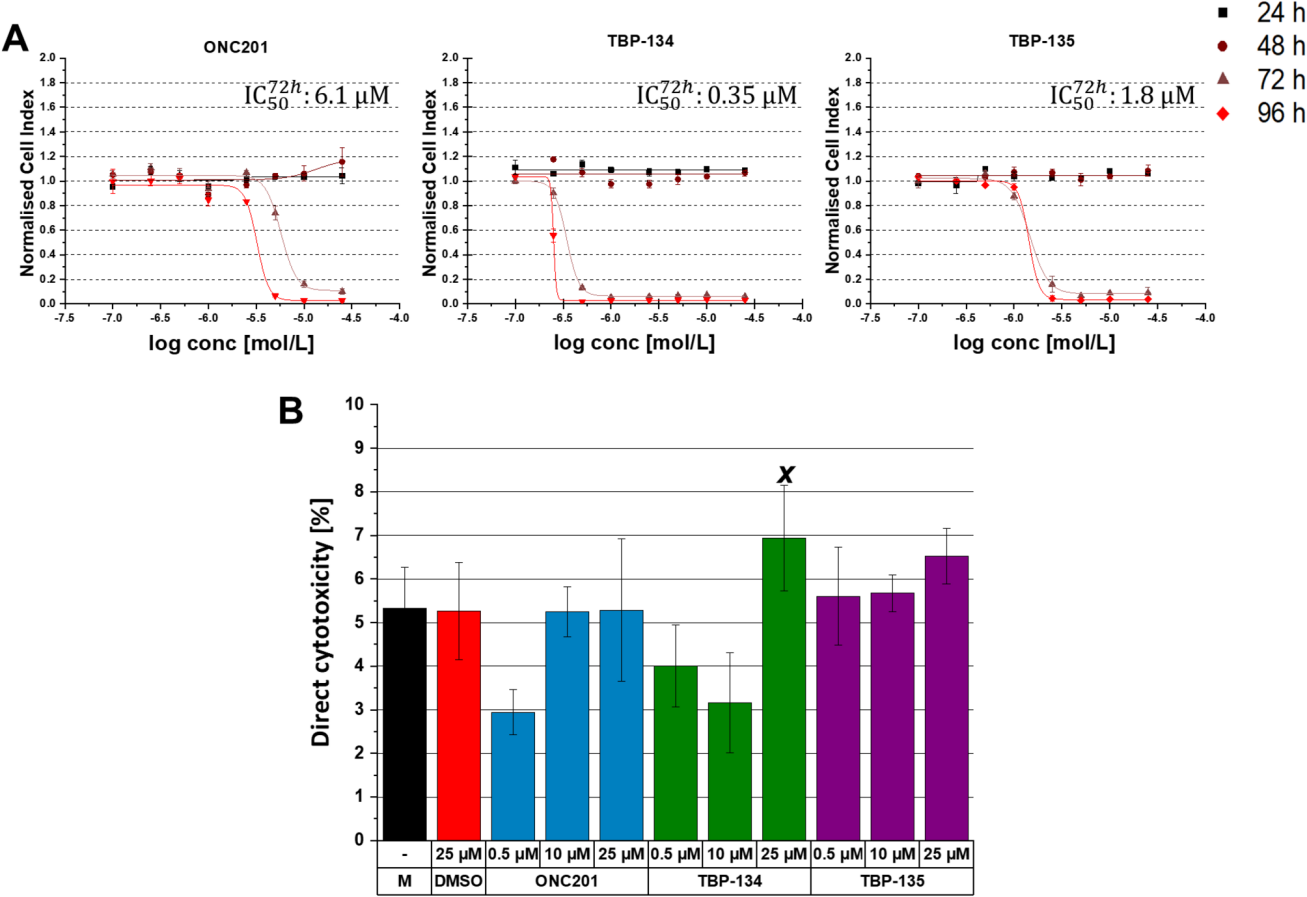
The effects of ONC201, TBP-134 and TBP-135 treatments on the cell viability and direct cytotoxicity of PANC-1 cells in vitro. (**A**) Dose-response curve of ONC201, TBP-134 and TBP-135 on PANC-1 cells. (**B**) The direct cytotoxicity (%) is determined with the following formula: $\:cytotoxicity\text{\%}=\:\frac{{LDH\:activity}_{treated}-{LDH\:activity}_{spontaneous}}{{LDH\:activity}_{maximum}-{LDH\:activity}_{spontaneous}}\:\text{x}\:100$ and was compared to the DMSO control. The data are presented as mean values ± standard deviation (SD) (*n* = 3). One-way ANOVA test followed by Fisher’s LSD post hoc test was used and the levels of significance are shown as x - *p* < 0.05.

Next, the half-maximal inhibitory concentration (IC_50_) values were determined for PANC-1 cells. The IC_50_ value indicates the concentration of the treatment required to reduce cell viability to 50% compared to the control-treated samples. No decrease in cell viability was detected after 24 and 48 h of treatment for any of the compounds (Fig. 1A). In the case of ONC201 the IC_50_ value was 6.1 µM after 72 h of treatment (Fig. 1A). While 72-hour treatment with TBP-134 and TBP-135, better IC_50_ values were determined as 0.35 and 1.8 µM, respectively. Interestingly, neither the parent molecule nor the two fluorinated derivates had IC_50_ value in the MIA PaCa-2 cell line.

In order to predict the probability of necrosis caused by the molecules, and consequently their inflammatory potential, the direct cytotoxicity % was established after 72-hour treatment. Neither molecule had a direct cytotoxic effect at 0.5 or 10 µM on PANC-1. However, treatment with 25 µM TBP-134 could increase it to 6.9% (1.3-fold) compared to the DMSO control (5.3%), while the other two molecules had no such effect (Fig. 1B).

### TBP-134 causes G2/M phase arrest in both PANC-1 and MIA PaCa-2 cells

**Fig. 2:**
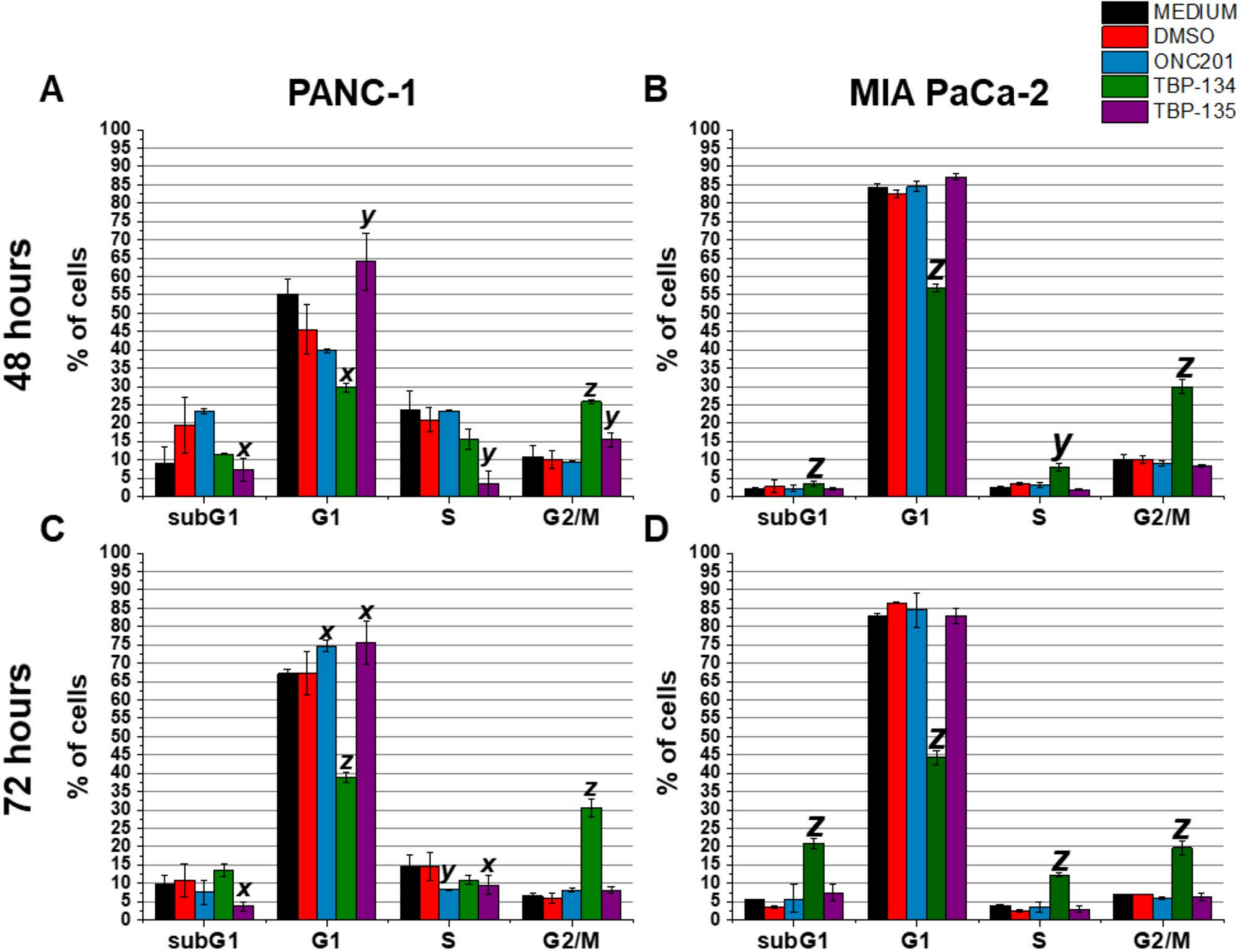
The effects of ONC201, TBP-134 and TBP-135 treatments on the cell cycle of PANC-1 and MIA PaCa-2 cells after 48- and 72-long incubation. Cell distribution (percentage of cells) in cell cycle phases is shown after (**A**,** B**) 48 and (**C**,** D**) 72 h of treatment with the compounds at 0.5 µM. The data are presented as mean values ± standard deviation (SD) (*n* = 2). Significance levels are calculated to the DMSO control and shown as x - *p* < 0.05; y - *p* < 0.01; z - *p* < 0.001 using one-way ANOVA test followed by Fisher’s LSD *post hoc* test.

The cell cycle assay was carried out on both PANC-1 and MIA PaCa-2 cells. After 48-hour treatment with 0.5 µM TBP-134 a strong G2/M phase arrest was detected on both PANC-1 and MIA PaCa-2 cell lines (Fig. 2A, B). While TBP-135 caused a G1 phase block in the cell cycle of PANC-1 cells, it did not affect MIA PaCa-2 cells. ONC201 did not have any influence on either cell’s cell cycle. However, the effect of the substances was most pronounced at 72 h (Fig. 2C, D, Supplementary Fig. S2). At 10 and 25 µM concentrations of ONC201 (2.1-fold), TBP-134 (2.2-fold), and TBP-135 (2.2-fold) had similar effects with a significant increase in cell number in the G2/M phase, accompanied by a significant decrease in the G1 phase (Supplementary Fig. S2).

Interestingly, the percentage of the sub-G1 phase of PANC-1 cells, representing the population of apoptotic bodies containing DNA fragments generated during apoptosis, did not increase significantly in either case. However, after treatment with 0.5 µM TBP-134, the subG1 phase of MIA PaCa-2 cells significantly increased from 5.6 to 20.8% (Fig. 2D).

### The molecules induce apoptotic cell death in PANC-1 cells

**Fig. 3:**
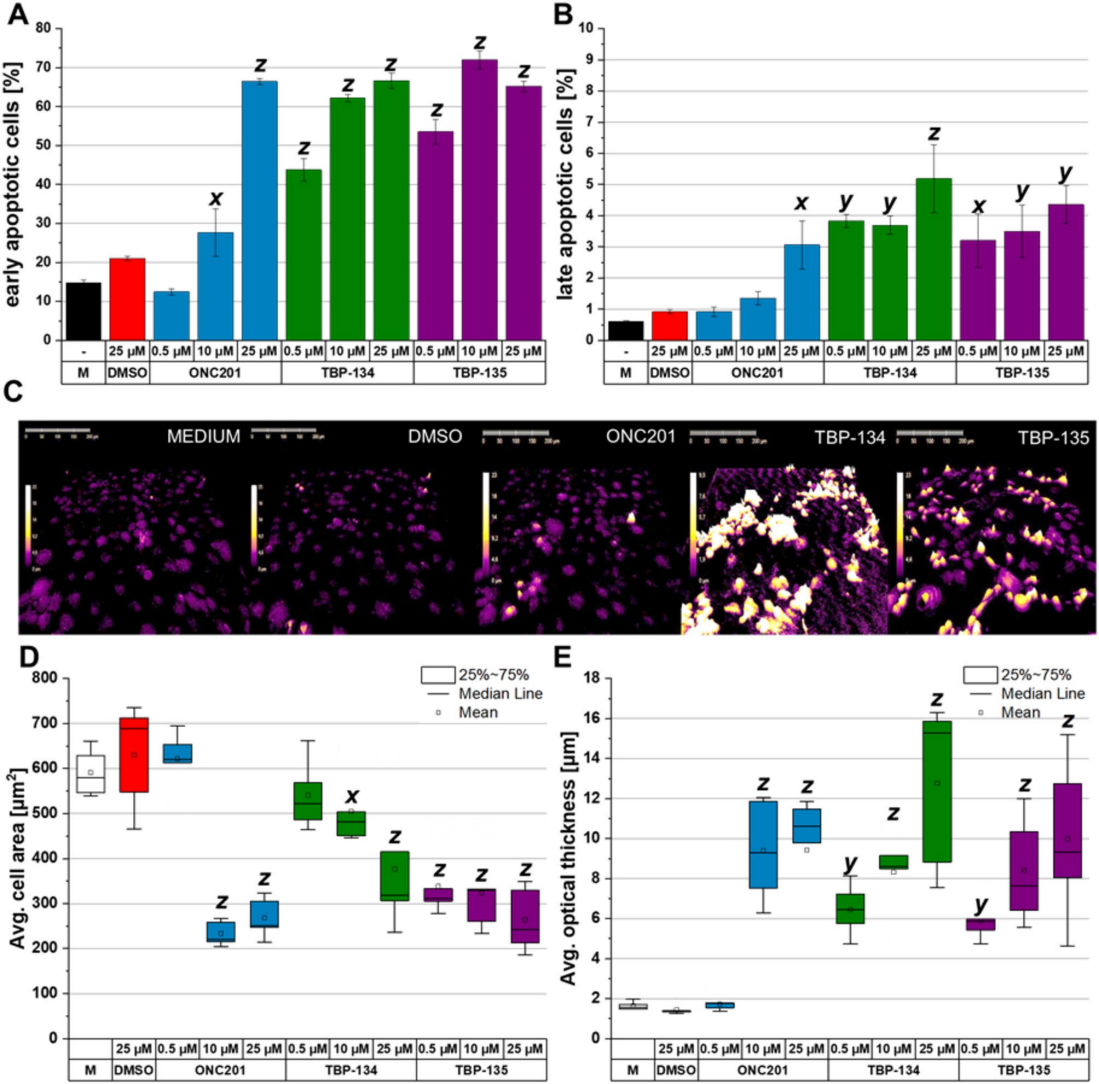
The apoptotic effects in PANC-1 cells after 72-hour long treatments with ONC201, TBP-134 and TBP-135. The percentage of (**A**) early- and (**B**) late apoptotic cells at 72 h shown in bar graphs. The data are presented as mean values ± standard deviation (SD) (*n* = 3). (**C**) The holographic microscopy images of PANC-1 cells after treatment with ONC201, TBP-134, and TBP-135 at 0.5 µM at 72 h. (**D**) The average cell area and (**E**) the average optical thickness after 72-hour treatment, are shown in box charts. The data are presented as mean values ± standard deviation (SD) (*n* = 5). M stands for Medium control. The data were normalised to the DMSO controls. Significance levels are shown as x - *p* < 0.05; y - *p* < 0.01; z - *p* < 0.001 using one-way ANOVA test followed by Fisher’s LSD *post hoc* test.

Based on the cell viability and cell cycle results, PANC-1 was the most sensitive to the molecules, thus the involved apoptotic pathways post-treatments with ONC201, TBP-134 and TBP-135 were determined on PANC-1. The time-dependent effects of the compounds were again seen in the case of the apoptosis assay. They had a mild effect after 24-hour treatment, but the apoptosis-inducing effect of the substances was most pronounced at 72 h (Fig. 3A, B, Supplementary Fig. S3). After 72-hour treatment, the cells were mainly in the early stage of apoptosis, at 0.5 µM TBP-134 and TBP-135 could significantly increase the ratio of AnnexinV+ and 7AAD- cell from 21.1 to 43.8% (by 2.1-fold) and 53.5% (by 2.5-fold), respectively (Fig. 3A). The two analogues also increased the ratio of late apoptotic cells from 0.9 to 3.8% (by 4.2-fold) and 3.2% (by 3.5-fold) at a concentration of 0.5 µM (Fig. 3B). In contrast, ONC201 had no apoptosis-inducing effect at 0.5 µΜ.

To monitor the morphometric changes in apoptosis, two parameters (cell area and optical thickness) were tracked for 72 h after treatment. There were no biologically relevant changes observed after 24 and 48 h of treatment with any compounds compared to the DMSO control (Supplementary Fig. S4). From the holographic microscopic images (Fig. 3C, Supplementary Fig. S4), it can be seen that for both medium and DMSO controls, the cells are flat, spread out and flattened against the surface, while after treatment, the cells were shrunk and rounded. The shrunken cells can be seen in the images as a light yellow colour, whereas in the case of a normal morphology (flattened out), they are dark purple. The average cell area decreased after 72-hour treatment with 0.5 µM TBP-135 to 338.5 µm^2^ (DMSO: 630 µm^2^; 1.9-fold) and increased the average optical thickness to 5.9 μm (DMSO: 1.45 μm; 4.1-fold) (Fig. 3D, E). For both parameters, it can be seen that the effect is concentration-dependent. In contrast, TBP-134 reduced the average cell area at a concentration of 25 µM to 376 µm^2^ (1.7-fold) but increased the average optical thickness to a greater extent at 0.5 µM to 12.8 μm (4.5-fold). At the same time, ONC201 at 0.5 µM did not affect cell morphology.

### The molecular background of the induced apoptosis in PANC-1 cells

**Fig. 4:**
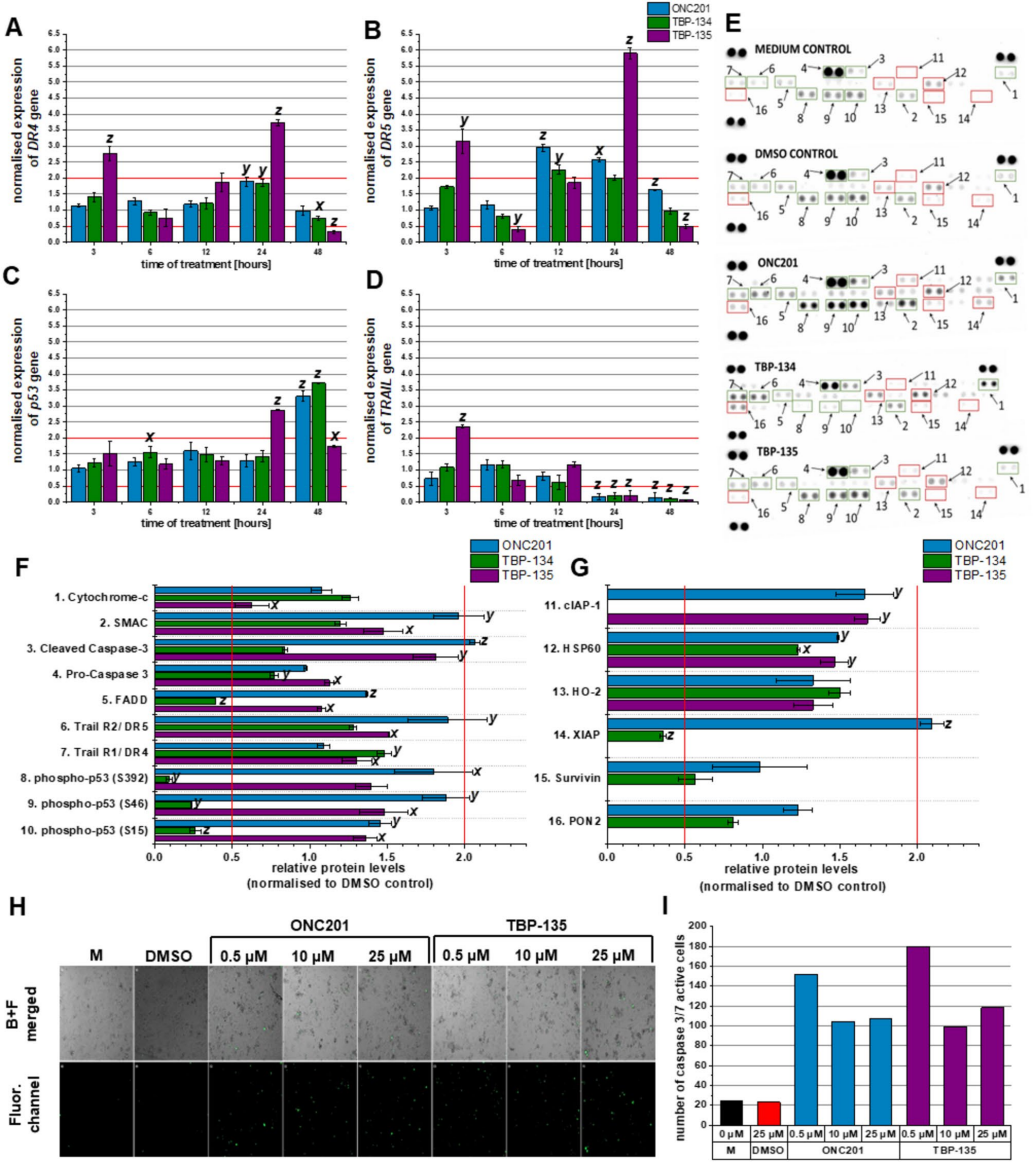
The mRNA expression of DR4, DR5, p53, and TRAIL genes in these cells over time, along with the assessment of the apoptotic proteins involved in PANC-1 cells following a 72-hour treatment with ONC201, TBP-134, and TBP-135. The normalised expression of (**A**) *DR4*, (**B**) *DR5*, (**C**) *p53* and (**D**) *TRAIL* genes are shown in column charts. (**E**) The proteome profile array blots of PANC-1 after treatment with 0.5 µM ONC201, TBP-135 and TBP-134 for 72 h. The dots for each protein are duplicates. The green squares indicate the pro-apoptotic proteins, while the red squares show the anti-apoptotic proteins quantified on the bar graphs. The relative (**F**) pro- and (**G**) anti-apoptotic protein levels are shown in bar charts. (**H**) Images of PANC-1 cells, including merged brightfield and fluorescence (FITC) channels as well as fluorescence (FITC) channel images, were captured using the Celldiscoverer 7 after 72 h of treatment with ONC201 and TBP-135. The green dots indicate the caspase 3/7 active cells. The abbreviation B + F merged refers to the merged image of the Brightfield and Fluorescence (FITC) channels, while Fluor. channel represents the Fluorescence (FITC) channel alone and M stands for Medium control. (**I**) The number of Caspase-3/7 active cells shown in column charts after 72 h of treatment with ONC201 and TBP-135. The red lines indicate the threshold of the changes in expression. The data were normalised to the DMSO control. The data are presented as mean values ± standard deviation (SD) (*n* = 3 – gene expression, *n *= 2 – proteom profile, *n* = 1 – caspase 3/7 activity). Significance levels are shown as x - *p* < 0.05; y - *p* < 0.01; z - *p* < 0.001 using one-way ANOVA test followed by Fisher’s LSD *post hoc* test.

Four genes (death receptors (*DR4, DR5*), *p53* and *TRAIL*) were selected to measure their expression at the RNA level. All three molecules upregulated the expression of *DR4* after 24 hours, but TBP-135 had the best effect. It increased the expression by 3.7-fold (Fig. 4A). ONC201 and TBP-134 upregulated the expression of *DR5* after 12-hour treatment (2.9-fold and 2.3-fold, respectively), while TBP-135 affected the gene expression after 24 hours, it increased the expression of *DR5* by 5.9-fold. (Fig. 4B). The expression of *p53* was induced later than the death receptors’, TBP-135 increased the expression after 24 h (2.9-fold), while ONC201 (3.3-fold) and TBP-134 upregulated it after 48 h. This gene was most affected by TBP-134 treatment, the expression increased by 3.7-fold (Fig. 4C). However, *TRAIL* induction was not detected at any of the time points we examined in the case of ONC201 and TBP-134 treatments. While TBP-135 affected the expression of *TRAIL* after 3-hour treatment, it showed a 2.4-fold increase (Fig. 4D).

We examined the changes in the apoptotic protein profile after treatment with the molecules at a concentration of 0.5 µM for 72 h. Our results show that 16 of the 35 proteins tested showed a significant change (increase or decrease). These include apoptotic proteins specific to the extrinsic and mitochondrial (intrinsic) pathways (Fig. 4E, Supplementary Fig. S5). After treatment with ONC201, the levels of several pro-apoptotic proteins significantly increased, such as cleaved caspase-3, FADD, DR5, SMAC, phospho-p53 (S392), phospho-p53 (S46) and phospho-p53 (S15) (Fig. 4F). Furthermore, this treatment could also increase the levels of some anti-apoptotic proteins, like cIAP-1, HSP60 and XIAP (Fig. 4G). Besides a significant increase in the expression of DR4 and HSP60, TBP-134 treatment could also decrease the levels of several pro- and anti-apoptotic proteins, such as pro-caspase 3, FADD, phospho-p53 (S392), phospho-p53 (S46), phospho-p53 (S15), XIAP (Fig. 4F, G). In addition, after treatment with TBP-134, the level of cIAP-1 was undetectable. Upon treatment with TBP-135, the levels of DR4, DR5, cleaved caspase-3, pro-caspase-3, SMAC, phospho-p53 (S46) and phospho-p53 (S15) were significantly increased, while the levels of cytochrome-c were significantly decreased. This treatment could also affect the expression of the anti-apoptotic proteins; the levels of cIAP-1 and HSP60 significantly increased, while they did not exert detectable change in XIAP and survivin levels (Fig. 4F, G).

In addition, the number of caspase-3/7 active cells was measured using CellEvent^TM^Caspase-3/7 Green Detection Reagent and imaged with Celldiscoverer 7. Based on our Proteome Profiler results, the kinetic changes of caspase-3/7 were monitored in ONC201 and TBP-135 treated PANC-1 cells. The substances were also the most effective in this experiment after 72 h (Fig. 4H, I, Supplementary Fig. S6). After 72-hour treatment, 0.5 µM, TBP-135 increased the number of caspase-3/7 active cells by 7.8-fold, while ONC201 increased them by 6.6-fold.

## Discussion

This study aimed to characterise the effects of the two fluorinated derivatives of ONC201 on two pancreatic adenocarcinoma cell lines (PANC-1, MIA PaCa-2) and three other tumour cell lines (A2058, EBC-1, COLO-205), and to determine the importance of the fluorination and its site. First, we measured the cell viability-reducing effect of the molecules on all five tumour cell lines at 3 different concentrations (0.5, 10, 25 µM) after 72 h of treatment. The results showed that all three molecules had significant antiproliferative effects on all tumour cell lines but with different potency. At the lowest concentration (0.5 µM), TBP-134 had the most significant effect, reducing the viability of tumour cell lines to approximately 50%.

A significant issue with the use of anti-tumour agents is their lack of selectivity, thus they can have the potential to damage healthy cells, leading to severe side effects. Therefore, normal human dermal fibroblast (NHDF) cells were also treated with the three molecules to determine their effect on healthy cells. This experiment showed neither molecule was cytotoxic to the NHDF cells; only TBP-134 had a slight antiproliferative effect. This means that these compounds do not significantly affect the viability of healthy cells. Therefore, the molecules exert their effect in a tumour-selective manner^18^. Furthermore, cardiotoxic side effects are common during treatment with anti-tumour agents and can persist as a chronic problem even after chemotherapy is completed^19^. For this reason, to assess the cardiotoxicity, the effects of the molecules on the viability of HL-1 cells were evaluated. After 72-hour treatment with all three molecules at the highest concentration (25 µM) the cell viability of the HL-1 cells remained above 80%. In a previous publication, we reported the strong cardiotoxic effects of daunorubicin and the cardioprotective properties of its conjugation with targeting peptides^20^. Based on that previous results we can state that ONC201 and its analogues spare the healthy cardiomyocytes and do not cause cardiotoxicity in vitro.

In the case of ONC201, the IC_50_ could be calculated as 6.1 µM after 72 h of treatment in PANC-1 cells. With the introduction of fluorine in the main structure, the efficacy improved in the case of the meta-substituted analogue, TBP-135, the IC_50_ value could be calculated as 1.8 µM. With the change of the site of fluorination, this effect was even more pronounced, as demonstrated by the markedly decreased IC_50_ value in the case of the para-fluorinated analogue, TBP-134. The IC_50_ was an order of magnitude lower compared to TBP-135. This result is consistent with the literature; Gillis et al. reported that with fluorination, the potency and permeability can be improved^16^.

Next, the direct cytotoxic effect was determined on PANC-1 cells to assess the potency of the molecules to cause necrosis and, therefore, inflammation^21^. After long-term treatment (72 h), neither ONC201 nor TBP-135 caused a significant cytotoxic effect, while TBP-134 at 25 µM could increase the direct cytotoxicity. This result shows that TBP-134 might cause direct cellular damage. However, it is important to highlight that it was only detected after treatment with 25 µM, which may distort results since IC_50_ of TBP-134 is nearly 100 times lower.

A crucial step in tumourigenesis is the disruption of the cell cycle regulation. Instead of cell division being arrested, abnormal cell division takes place^22^. Hence, a major starting point in anti-tumour therapies is to use a drug that can stop the cell cycle and induce apoptosis afterwards. After 48 h of treatment with 0.5 µM TBP-134, G2/M phase arrest was detected, coupled with a significant decrease in the ratio of cells in the G1 phase, therefore forcing the cells to stop their cell division. On the other hand, ONC201 and TBP-135 could arrest the cell cycle only in higher concentrations. TBP-134 could also act in the same manner on MIA PaCa-2 cells causing G2/M phase arrest after 48-hour treatment, while the other two molecules did not have any cell cycle-altering effect even after 72 h. G2/M phase arrest is important in tumour therapy, not only because it stops cell proliferation but also because it sensitises tumour cells to other anticancer drugs^23^. Several G2/M inhibitors have already been characterised, including cyclin-dependent kinase inhibitors such as flavopiridol^23^. The combination of flavopiridol and docetaxel was evaluated in a phase 2 clinical trial in refractory metastatic pancreatic cancer, which unfortunately ended with unsatisfactory results^24^.

Once the cell cycle has been arrested, it is ideal for the cells to undergo apoptotic cell death. Regarding the sensitivity of the different cell lines to the investigated molecules, PANC-1 seemed to be the right model cell to characterise the differences in the mechanism of action of the differently fluorinated molecules, therefore the following experiments were conducted only on PANC-1 cells. Induction of apoptosis was seen on PANC-1 cells after 24 h of treatment with all three molecules at 10 µM. However, the most substantial effect was seen after 72 h of treatment. At this time, the majority of the cells were in the early phase of apoptosis, while a smaller proportion had already entered the late apoptotic stage. This result was consistent with the holographic images of the cells, which showed the characteristic morphological changes during apoptotic cell death, which means reduced cell area and increased optical thickness^25,26^. These results show that the fluorinated analogues of ONC201 can induce apoptosis to a greater extent than the parent molecule. The two molecules showed similar effects, except for their IC_50_ values, based on these measurements, so the next step was to determine the mechanism of action of TBP-134 and TBP-135, focusing on the molecular background of the induced apoptosis.

One way to initiate programmed cell death is by activating it with TRAIL through the extrinsic pathway. This has long been discussed as a new approach to tumour therapy^27^. TRAIL-based tumour therapy has been the subject of many publications in recent years, and two approaches can be distinguished: treatment with recombinant human TRAIL (rhTRAIL), and therefore, activation of TRAIL receptors (DR4, DR5) or induction of TRAIL up-regulation^28^. One rhTRAIL, dulanermin, has already been used in a clinical trial for metastatic colorectal cancer combined with modified FOLFOX6 and bevacizumab^29^. Unfortunately, it has also been shown that resistance to this therapy can easily develop in the case of pancreatic ductal adenocarcinoma^30,31^. It has been suggested by previous papers that ONC201 may induce the expression of TRAIL. Contrary to the data found in the literature, in our experiments, ONC201 did not cause a significant upregulation of *TRAIL* at the mRNA level, whereas TBP-135 did after 3 h of treatment^9^. In addition, TBP-135 increased the expression not only of *TRAIL* but also of its receptors (*DR4, DR5*) and *p53*. TBP-135 upregulated the expression of *p53* at 24 h at the mRNA level, but ONC201 and TBP-134 had a more significant effect at 48 h. The expression of *DR4* and *DR5* was elevated both at mRNA and protein levels after treatment with ONC201 and TBP-135. Notably, TBP-135 significantly upregulated the expression of both death receptors after 3-hour treatment. Wajant et al. found that the two death receptors could be activated differently^32,33^. DR5 can only be activated by the membrane-bound form of TRAIL, while DR4 activates by soluble and membrane-bound forms. Furthermore, Lemke et al. reported that in the case of PDAC cells, DR4 is more responsible for initiating apoptosis^34^. This finding aligns with our results, which showed that TBP-134 may act predominantly through DR4, while the other two molecules act through DR5. Previously, we reported that the ONC201 and bortezomib combination acts synergistically on A2058 metastatic melanoma cells, presumably through DR5 upregulation^35^. At the same time, Zhang et al. reported that ONC201 could sensitise PANC-1 cells to gemcitabine^9^. Our results on the death receptor and TRAIL levels, combined with findings in the literature, suggest that even the fluorinated analogues of ONC201 may have the potential to exert synergistic effects with other cytotoxic chemotherapeutic agents. In this case, using reduced amounts of the drugs would result in more tolerable side effects, improving patients’ quality of life.

It is also worth mentioning that an unexpected significant decrease was seen after treatment with all three molecules in the expression of *DR4* and *DR5* after 48 h of treatment and *TRAIL* after 24 h of treatment, which can be explained by the degradation of mRNA at these time points as these three proteins are among the initiators of apoptosis. Hence, as time goes on, the apoptotic process is further advanced, and the expression of downstream effector molecules will be upregulated. The proteome profiling results showed that ONC201 and TBP-135 induced apoptosis by simultaneously activating the extrinsic- and intrinsic pathways. While ONC201 significantly increased the level of an anti-apoptotic protein, XIAP, it also increased the level of SMAC, which is the endogenous inhibitor of XIAP, allowing the process of apoptosis to proceed uninterrupted^36^. It is worth noting that TBP-135, in contrast to ONC201, significantly reduced the levels of anti-apoptotic proteins, or several proteins were undetectable after treatment (e.g. XIAP, survivin). While the TBP-134 treatment increased the expression of DR4 at the protein level, it also caused a significant decrease in the levels of some anti-apoptotic proteins (cIAP-1, XIAP). Contrary to the mRNA gene expression results, it decreased the levels of activated forms of p53 and did not affect the levels of cleaved caspase 3. This discrepancy between the results of the mRNA gene expression and the proteome profiling could be caused by the difference in the used concentrations and the different time points. Also, TBP-134 treatment may result in a different phosphorylation pattern of p53, which was not detected by the proteome profiler membrane.

A possible mechanism for the p53-independent activation of the intrinsic apoptotic pathway is the hyperactivation of the mitochondrial ClpP protein^37^. It has been previously reported that the ONC201 molecule can directly bind to this enzyme, leading to its hyperactivation^10^. Although our studies did not specifically investigate whether the fluorinated analogues possess this ability, we hope that this may also contribute to their increased efficacy.

A kinetic assay was performed to prove the increased level of cleaved caspase 3 functionality in the case of ONC201 and TBP-135 treatment. This assay could determine the increasing number of cells that were caspase 3/7 positive. The result of this experiment aligned with the proteome profile result: after 72 h of treatment, both molecules increased the number of caspase 3/7 active cells.

**Fig. 5:**
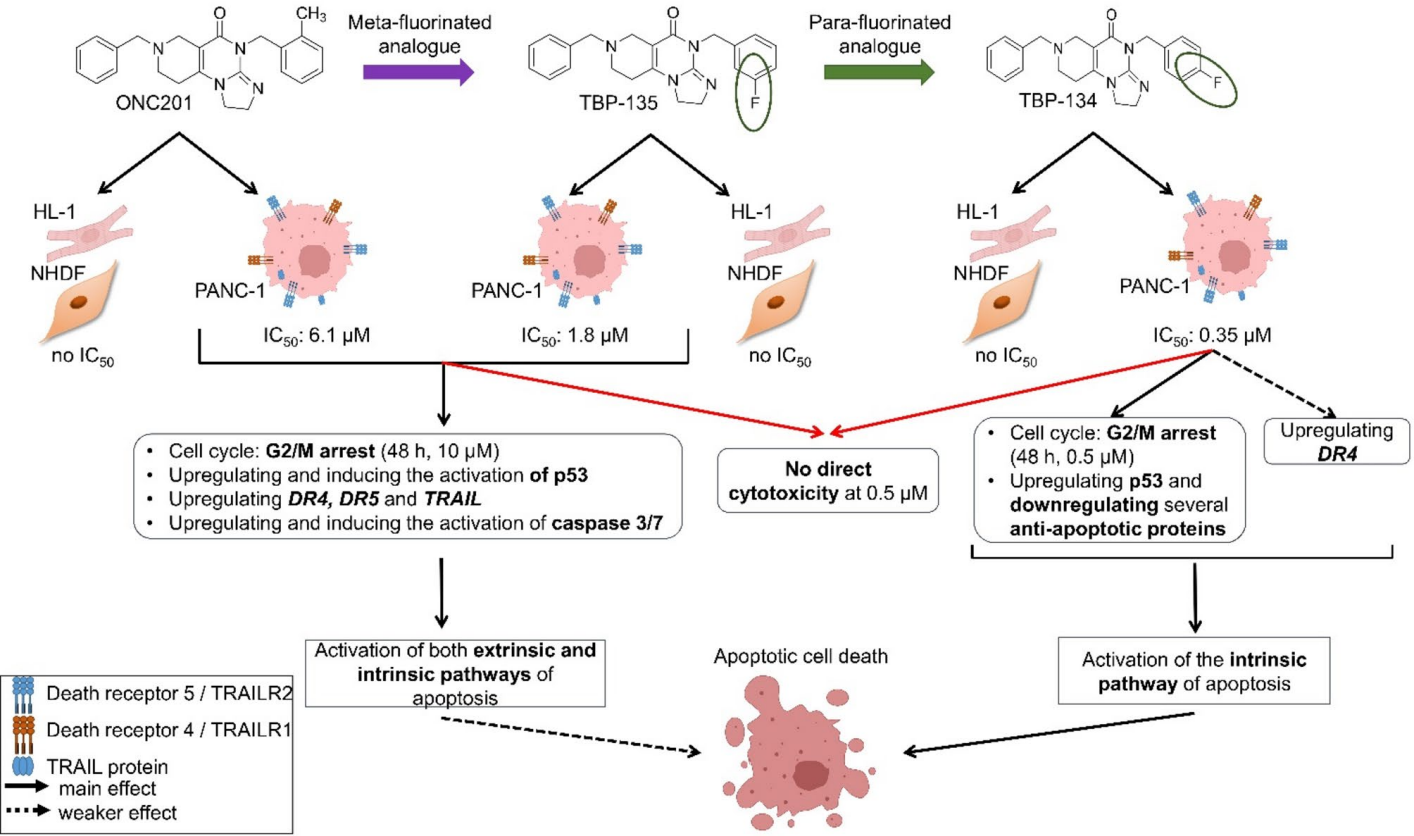
The graphical summary of the anti-tumour effect of the ONC201 and the two fluorinated analogues on PANC-1 and NHDF cells.

The results of this study show that the two derivatives exert their anti-tumour effect on PANC-1 cells in slightly different ways (Fig. 5). While introducing fluorine into the structure of the demethylated version of the parent molecule (ONC201) can improve potency, the mechanism of action remains the same, activating both apoptosis pathways. This was observed using the meta-fluorinated analogue, TBP-135. On the other hand, changing the fluorination site not only increased potency but altered the molecular background of the induced apoptosis. The para-fluorinated analogue, TBP-134, is thought to act mainly through the intrinsic pathways of apoptosis, with less involvement of the extrinsic pathway, mainly through DR4. Due to para-fluorination in TBP-134, an altered steric effect could arise within the molecule, and a slight shift in polarity relative to the meta-fluorinated analogue, TBP-135, may also be observed^38^. This variation in the electron distribution may account for TBP-134, lowering the induction of the TRAIL-dependent extrinsic apoptotic pathway while demonstrating an increased capacity to activate the intrinsic apoptotic pathway through p53 activation. However, to establish this, further investigations are required. Altogether, the site of fluorination is crucial in developing derivatives for anti-tumour therapies. As Álvarez-Buílla et al. reported in the case of many drugs with quinazolinone structure, the site of fluorination can influence the mechanism of action, and the type of effect^39^. Our result is aligned with the trend in drug development and the literature data on medicinal chemistry research. The number of FDA-approved fluorinated drugs is increasing yearly, demonstrating that this approach is widely utilised and recognised for enhancing a drug’s potency and modifying its physicochemical properties^40^. For example, fluorination has shown significant benefits in the case of the fluoroquinolone antibiotic family and fluorinated corticosteroids^41,42^.

## Conclusions

In conclusion, this study effectively demonstrated that the fluorinated derivatives of ONC201 exhibit significant cell viability-reducing effect in PANC-1 cells, with TBP-134 showing the greatest efficacy at 0.5 µM. Importantly, the compounds did not exhibit significant cell viability-decreasing effect towards healthy human dermal fibroblast cells or cardiomyocytes, suggesting a favourable tumour-selective profile. After 72-hour treatment the IC_50_ values were determined on PANC-1 cells as the following: ONC201 (6.1 µM) > TBP-135 (1.8 µM) > TBP-134 (0.35 µM). Notably, 0.5 µM TBP-134 induced G2/M phase arrest in the cell cycle of both PANC-1 and MIA PaCa-2 cells after 48 h of treatment. While TBP-135 and ONC201 showed this effect only at higher concentrations on PANC-1 cells. All further studies were conducted on PANC-1 cells based on its sensitivity for the drugs. Although none of the imipridones showed direct cytotoxic (necrosis-inducing) effects on PANC-1 cells at a concentration of 0.5 µM after 72 h, all these molecules induced apoptosis within 24 h, but their optimal effects were observed after 72 h. 0.5 µM TBP-134 and TBP-135 increased the early to late apoptotic cell ratio at 72 h. This was confirmed by holographic microscopy images showing decreased cell area and increased optical thickness. The mechanisms of action differed between the molecules: ONC201 induced both extrinsic and intrinsic pathways of apoptosis, although to a lesser extent than TBP-135. Both molecules exerted their apoptosis-inducing effects primarily through DR5. In addition, both ONC201 and TBP-135 increased the number of caspase-3/7 active cells after 72 h of treatment at 0.5 µM, with TBP-135 showing a superior effect. In contrast, while TBP-134 predominantly activated the intrinsic pathway, it also exhibited a minor effect through DR4. Despite activating mainly the intrinsic pathway, TBP-134 proved to be the most potent of the three compounds in our study. Ultimately, these results suggest that the site of fluorination is crucial not only for potency but also for the mechanism of action of these compounds. However, these findings are preliminary and require validation through in vivo xenograft studies to establish greater confidence in the efficacy and potency of these compounds^43^.

Overall, TBP-134 has shown greater efficacy in our experiments and could be considered as a very promising lead for structural optimisation in the imipridone family to identify further representatives featuring enhanced potency in the treatment of pancreatic tumours.

## Materials and methods

### Substances

ONC201 and its derivates were provided by Prof. Csámpai (Institute of Chemistry, Eötvös Loránd University, Budapest, Hungary), which were dissolved in dimethyl sulfoxide (DMSO; AppliChem GmbH, Darmstadt, Germany) (stock solution: 10^−2^ M). The stock solution was aliquoted and kept at -80 °C, and for each experiment, we prepared fresh dilutions of the stock solution in a cell culture medium. The final concentration of DMSO was < 1 v/v% for each treatment group.

**Fig. 6:**
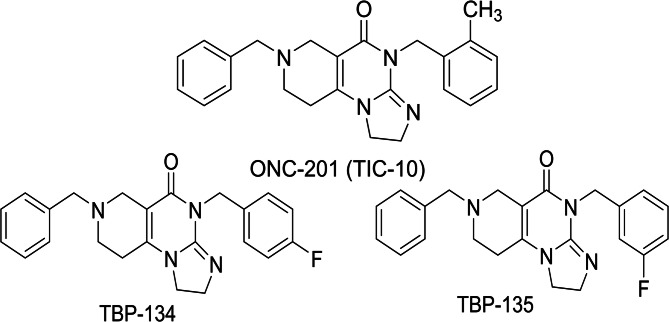
The molecular structures of ONC201 (TIC-10), TBP-134 and TBP-135.

The fluorinated ONC201 analogues para-fluorinated TBP-134, and meta-fluorinated TBP-135 (Fig. 6) were accessed via the convergent synthetic pathways as we have described and characterized in our PCT^44^.

### Cell culturing

For our experiments, we used two pancreatic adenocarcinoma cell lines PANC-1 and MIA PaCa-2 obtained from the European Collection of Authenticated Cell Cultures (ECACC, Salisbury, UK) to evaluate the effects of ONC201 and its derivatives on cell physiological parameters. Three other tumour cell lines with high mortality rates were used to determine the potential tumour selectivity of the compounds, these were COLO-205 (colorectal adenocarcinoma), and A2058 (metastatic melanoma), which were obtained from the European Collection of Authenticated Cell Cultures (ECACC, Salisbury, UK) and EBC-1 (lung squamous cell carcinoma) purchased from the Japanese Collection of Research Bioresources Cell Bank (JCRB Cell Bank, Osaka, Japan). Normal human dermal fibroblast cells (NHDF, PromoCell, Heidelberg, Germany) were also used to test the molecules’ effect on healthy cells. Immortalised mouse cardiomyocyte cell line (HL-1) obtained from Merck (Darmstadt, Germany) was used to determine the cardiotoxic effect of the drugs.

PANC-1 (87092802 ECACC) cells, MIA PaCa-2 cells (85062806 ECACC) and EBC-1 (JCRB0820 JCRB Cell Bank) cells were cultured in DMEM (Lonza Group AG, Basel, Switzerland), A2058 (91100402 ECCAC) cells were cultured in RPMI (Lonza Group AG, Basel, Switzerland), COLO-205 (87061208 ECCAC) cells were cultured in High glucose DMEM (Lonza Group AG, Basel, Switzerland).

The basal media were supplemented with 10% fetal calf serum (Biosera, Nuaille, France), 1% L-glutamine (Lonza Group AG, Basel, Switzerland) and 1% penicillin/streptomycin (Sigma Ltd. St. Louis, MO, USA). The medium for EBC-1 was further completed with 1% non-essential amino acids (Sigma Ltd. St. Louis, MO, USA) and 1% sodium pyruvate (Sigma Ltd. St. Louis, MO, USA). All cells were stored in liquid nitrogen after purchase and used for up to 6 months after thawing.

The NHDF cell line obtained from PromoCell (*PC-C-12300) was cultured in a Fibroblast Growth Medium supplemented with the Supplement Mix recommended by the distributor (PromoCell, Heidelberg, Germany). After purchase, the cells were stored in liquid nitrogen and used for 3–5 passages after thawing.

HL-1 cells (Lot: RD1601001, SCC065) were cultured in Claycomb Medium (Merck, Darmstadt, Germany) supplemented with 10% HL-1 screened fetal calf serum (Merck, Darmstadt, Germany), 1% L-glutamine (Lonza Group AG, Basel, Switzerland), 1% penicillin/streptomycin (Sigma Ltd. St. Louis, MO, USA) and 0.1 mM norepinephrine (± arterenol, Sigma Ltd. St. Louis, MO, USA, 100x stock in 30 mM L-ascorbic acid ). The cells were passaged using coated cell culture flasks. The coating was done using 0.02% gelatine solution (Sigma Ltd. St. Louis, MO, USA) containing 5 µg/mL fibronectin (Merck KGaA, Darmstadt, Germany), incubated overnight at 4 ◦C. Before use, the remaining gelatine-fibronectin solution was discarded, and the surface was allowed to dry for 10 min.

### Measuring cell viability and IC_50_

The exact IC_50_ value was calculated by using OriginPro 8.0 software by plotting viability values as a function of log concentrations of the molecules used, fitting a curve to the points (Sigmoidal Fitting/Dose Response Curves), and reading the concentration value corresponding to a 50% reduction in viability. We have tested the compounds in a concentration range from 250 nM to 50 µM.

The xCELLigence SP (Agilent, Santa Clara, CA, USA) was used to investigate the effects of the molecules on the viability of PANC-1 cells. The operation of xCELLigence SP is based on a real-time impedimetric measurement technique. The measurements performed by this technique are capable of quantitatively characterising cell proliferation, morphological changes and the extent of cell proliferation^45^. First, a baseline was established with a cell-free culture medium for 1 h at 1-minute intervals. Cells (10^4^ cells/well/200 µL) were then added to the system, and cell adhesion was monitored by detecting the impedance for 24 h at every 15 min. The changes in impedance were expressed as a relative and dimensionless parameter, the Cell Index (CI). At the end of the 24 h, the drugs to be tested were added to the system, and the CI was recorded every 5 min for 24 h and then every 15 min for 72 h.

The alamarBlue assay (Thermo Scientific, Waltham, MA, USA) was used to test the viability of MIA PaCa-2, A2058, EBC-1, COLO-205, NHDF and HL-1 cells^46^. Cells were plated on a 96-well culture plate at different concentrations: 1 × 10^4^ cells/well in 200 µL for tumour cell lines, 2.5 × 10^3^ cells/well in 200 µL for NHDF and 4 × 10^4^ cells/well in 100 µL for HL-1 cardiomyocytes. After 24 h of seeding the tumour cell lines and NHDF cells, the cells were treated with ONC201, its derivatives and control solutions. After 72 h of seeding the HL-1 cells to gelatin-fibronectin-coated plates with ascorbic acid-free media, an additional 100 µL of fresh media was added before the cells were treated with the drugs. After 24, 48 and 72 h of incubation, alamarBlue (0.15 mg/mL resazurin, Thermo Fisher Scientific, Waltham, MA, USA) was added to the cells and after 5 h of incubation, the samples were measured using Fluoroskan FL Microplate Fluorometer and Luminometer (Thermo Scientific, Waltham, MA, USA) (λ_ex_: 530–560 nm, λ_em_: 590 nm).

Cell culture medium and DMSO (< 1 v/v%) were used as controls in both viability experiments and all the following measurements. The DMSO treatment was always the control for the highest tested concentration. Unless otherwise stated, the experiments were performed in triplicates, and the results were normalised to the DMSO control.

The incubation time (24, 48 and 72 h) and test concentrations (0.5, 10 and 25 µM) of further experiments were determined on the IC_50_ values measured on PANC-1 cells.

### Measuring cellular cytotoxicity

To assess the direct cytotoxicity, the CyQUANT™ LDH Cytotoxicity Assay (Invitrogen Corporation, New York, NY, USA) was used, which is a colourimetric assay. It is based on the detection of lactate dehydrogenase (LDH) activity. When there is damage to the plasma membrane, LDH is released into the cell culture medium, where it can be detected. First, 5 × 10^3^ cells/well/100 µL concentration of cells were seeded in a 96-well plate, and after 24 h of incubation, the cells were treated with the molecules at concentrations of 0.5, 10 and 25 µM. After 72 h of treatment, 50 µl of the media from each sample was pipetted to a new 96-well plate. Then, the necessary steps were carried out according to the manufacturer’s instructions. Absorbance was measured for each well using a plate reader (Multiskan MS, Labsystems, Helsinki, Finland) at 492 and 680 nm. The % cytotoxicity was then calculated according to the manufacturer’s instructions. Three parallel samples were analysed in each treatment group.

### Cell cycle analysis

For cell cycle assays, the DNA content of cells was measured on a flow cytometer using the fluorescent DNA intercalating dye propidium iodide (PI)^47^. Sample preparation and data evaluation are quite similar to our previous work^48^. First, cells were plated in 24-well culture plates at a concentration of 1.5 × 10^5^ cells/well/450 µL. 24 h after seeding, cells were treated with the drugs in a concentration of 0.5, 10 and 25 µM. At 48 and 72 h time points, cells were passaged by adding 150 µl PBS and adding 100 µl trypsin-EDTA followed by centrifugation (5 min, 300 g). The supernatant was discarded, and cells were resuspended in 70% ethanol at -20 °C and allowed to stand at room temperature for 30 min, followed by incubation for a further 24 h at -20 °C. Then, the cells were centrifuged again (5 min, 300 g) to remove the ethanol. Cells were then resuspended in citric acid/ disodium hydrogen phosphate buffer (pH = 7.8) containing 100 µg/mL RNase A (Sigma Ltd. St. Louis, MO, USA). Immediately before measurement, 6 µL of PI (Sony Biotechnology, Weybridge, UK) was added to each sample. In the flow cytometric measurement (BD FACSCalibur, Becton–Dickinson, San Jose, CA, USA), the number of measured cells was 2.5 × 10^4^ cells/min; in each case, 2 parallel samples were analysed, and PI was detected using FL-2 (585/42). The results were evaluated using Flowing Software 2.5.1 (Turku Centre of Biotechnology, Turku, Finland).

### Defining the ratio of early and late apoptotic cells

For apoptosis measurements, Annexin-V positive/7AAD (7-Aminoactinomycin D) negative, early apoptotic and Annexin-V/ 7AAD double positive, late apoptotic cells were differentiated using a BD FACSCalibur (BD FACSCalibur, Becton-Dickinson, San Jose, CA, USA) flow cytometer^49,50^. First, 7 × 10^4^ cells/well/900 µL concentration of cells were seeded in a 24-well plate, and after 24 h of incubation, cells were treated with the molecules at concentrations of 0.5, 10 and 25 µM. After 24, 48, and 72 h treatment, cells were harvested with 300 µL TrypLE (Sigma Ltd. St. Louis, MO, USA). Fresh cell culture medium was then added to stop the effect of TrypLE and the harvested cells were centrifuged for 5 min (300 g). Then, the supernatant was removed, and cells were resuspended in 300 µL of Annexin binding buffer (Sony Biotechnology, Weybridge, UK). For labelling, 3–3 µL of Annexin-V-FITC (Sony Biotechnology, Weybridge, UK) and 7AAD (Sony Biotechnology, Weybridge, UK) were added, followed by 10 min incubation at room temperature in the dark. For flow cytometric analysis (BD FACSCalibur, Becton–Dickinson, San Jose, CA, USA), a minimum of 10^4^ cells/sample was measured. Annexin-V was detected using the FL-1 channel (530/30), and 7AAD was detected using the FL-3 channel (670 LP). In each treatment group, 3 parallel samples were analysed. The results were evaluated using Flowing Software 2.5.1 (Turku Centre of Biotechnology, Turku, Finland).

### Tracking the morphometric changes of apoptosis

To follow the morphometric changes, a holographic transmission microscope (HoloMonitor M4; Phase Holographic Imaging AB, Lund, Sweden) was used, which uses a laser to produce 3D images of the cells^51,52^. This in vivo imaging technique allows us to monitor cell morphological changes in real-time. First, the cells were seeded at a concentration of 3.5 × 10^5^ cells/flask/4000 µL cell culture medium. After 24 h of seeding, the cells were treated with the drugs at a concentration of 0.5, 10 and 25 µM, and images were taken at 24, 48 and 72 h after the treatment. At least 5 images were taken from each culture dish, taking care to assess different fields of view. These images were evaluated using the microscope software (Hstudio M4). We assessed the average area and the average optical thickness of the cells.

### Measurement of changes in apoptotic gene expression

Based on the literature on the mechanism of action of ONC201 and our apoptotic protein profiling results, we selected 4 genes to measure their expression at the RNA level. These were the death receptors DR4 and DR5, TRAIL and p53. Sample preparation and data evaluation are very similar to those performed in one of our previous papers^35^. First, the cells were plated at 0.5-1 × 10^6^ cells/flask/4500 µL cell culture medium. After 24 hours of seeding, we treated the cells with the substances in a concentration of 25 µM. After 3-, 6-, 12-, 24- and 48-hour incubation, total RNA was isolated with the RNeasy kit (Qiagen, Hilden, Germany) according to the manufacturer’s manual. The treatment with the highest concentration (25 µM) was chosen to detect early changes in gene expression (3–12 hours). After the isolation, we measured the RNA concentration using the NanoDrop-1000 spectrophotometer (Thermo Fisher Scientific, Waltham, MA, USA). RNA was then reverse transcribed into cDNA at a concentration of 1000 ng/20 µL using the SensiFAST™ cDNA synthesis kit (Bioline Reagents Ltd., London, UK). We used Sso Advanced Universal SYBR Green Supermix (BioRad, Hercules, CA, USA) for the amplification in 20 or 10 µL final volume for the 96- or 384-well PCR plate, respectively. The measurement was performed using the CFX96 or CFX384 Touch™ Real-Time PCR system with the BioRad CX Maestro software (BioRad, Hercules, CA, USA). We used three technical replicates with all the samples for the same target genes, and ’no template’ controls (NTC) were used for each primer. We used pre-designed gene-specific primers, which were the following: DR4 (*TNFRSF10A*, unique assay ID: qHsaCID0018590); DR5 (*TNFRSF10B*, unique assay ID: qHsaCED0036477); p53 (*TP53*, unique assay ID: qHsaCED0045022); TRAIL (*TNFSF10*, unique assay ID: qHsaCED0036477); glyceraldehyde-3-phosphate dehydrogenase (*GAPDH*, unique assay ID: qHsaCED0038674); TATA-box binding protein (*TBP*, unique assay ID: qHsaCID0007122). The 2^−(ΔΔCt)^ method was used to calculate the changes in gene expression. GAPDH and TBP were used as housekeeping genes for normalisation. At least a 2-fold change is considered upregulation, whereas a 0.5-fold change indicates downregulation.

### Analysis of apoptotic protein expression

To map changes in the expression of apoptotic proteins, we used the Human Apoptosis Array kit (R&D Systems, Minneapolis, MN, USA), which provides qualitative and semi-quantitative information on the expression of 35 apoptotic proteins. Total protein was first isolated from cells after 72 h of treatment at a concentration of 0.5 µM. We chose the lowest concentration (0.5 µM) to ensure that a high percentage of cells were in active apoptosis and to obtain sufficiently measurable protein levels. One confluent culture dish (1.5 × 10^6^ cells/flask) was used for protein isolation for each treated sample and control. Sample preparation and data analysis are very similar to those described in this article^53^. First, the medium was aspirated from the cells into a 10 mL tube, and then the cells were washed with PBS and digested with trypsin-EDTA from the bottom of the culture dish. This was followed by centrifugation for 3–5 min (300 g), and after aspiration of the supernatant, the cells were resuspended in 1 mL PBS/tube and pipetted into an Eppendorf tube. The samples were then centrifuged again for 5 min (300 g) and washed again with PBS (1 mL). This washing step was repeated twice. After the last wash, the PBS was carefully drained and 250 µL of Lysis Buffer 17 (Lysis Buffer 17, R&D Systems, Minneapolis, MN, USA) containing 10 µg/mL aprotinin, 10 µg/mL leupeptin, 10 µg/mL pepstatin (Merck, Darmstadt, Germany) was added to the cells and incubated at 4 °C for 20 min. At the end of the incubation period, cells were centrifuged at 4 °C at 20,000 g for 20 min. The supernatant containing the isolated proteins was carefully aspirated and pipetted into new Eppendorf tubes. After isolation, total protein concentration was determined using the colourimetric Micro BCA Protein Assay Kit (Thermo Scientific, Waltham, MA, USA). An 8-member two-fold serial dilution series (0–200 µg/mL) of BSA (bovine serum albumin) in PBS was required to determine the protein concentration accurately. The required reagent mixture was prepared according to the manufacturer’s instructions (50% reagent A, 48% reagent B and 2% reagent C). Subsequently, we added 100 µL of reagent mixture to our samples (100 µL) and calibration dilutions (100 µL). After 1 h of incubation at 60 °C and cooling down to room temperature, we transferred the samples to a 96-well microplate, which was read at dual wavelengths (540 nm and 620 nm) using an ELISA reader (Multiskan MS, Labsystems, Helsinki, Finland). The absorbance results of the 620 nm read were subtracted from the results obtained at 540 nm.

After determining the exact protein concentration, the Human Apoptosis Array Kit was used to determine the apoptotic protein profile. To do this, we first calculated the volume of 225 µg of protein for each sample. Then, the proteins were added to the membranes, and the necessary steps were performed according to the manufacturer’s manual. Images of the protein-labelled membranes were taken using the Bio-Rad Chemidoc XRS + instrument (BIO-RAD, Hercules, CA, USA), and the results were evaluated using ImageLab software (BIO-RAD, Hercules, CA, USA) and MS Excel. Results were expressed as normalised pixel density, a ratio calculated using the density of reference points on the membrane. A change of at least 2-fold is considered for upregulation, while a change of 0.5-fold indicates downregulation.

### Detection of caspase-3/7 activity

CellEvent^TM^Caspase-3/7 Green Detection Reagent (ThermoScientific, Waltham, MA USA) was used to measure caspase-3/7 activity and images were captured using ZEISS Celldiscoverer 7 (Carl Zeiss AG, Jena, Germany). Cells were initially plated at 10^5^ cells/mL in black 96-well plates with optical bottom. After 24 h of seeding, they were treated with ONC201 and TBP-135 at 0.5, 10 and 25 µM concentrations. CellEventCaspase-3/7 was then added, and the measurement was performed according to the manufacturer’s instructions. A kinetic setup was used to detect changes in the number of caspase-3/7 positive cells. Images were taken using Celldiscoverer 7, one position/well, using the same field of view for each well at each time point. The samples were imaged using 5 × Plan-Apochromat 5×/0.35 NA objective with 2× tube lens (Carl Zeiss AG, Oberkochen, Germany) using brightfield (exposure time: 10 ms) and FITC channel (λex: 495 nm, λem: 519 nm, exposure time: 500 ms). Images were analysed using ZEN blue 2.6 software (Carl Zeiss AG, Oberkochen, Germany).

### Statistical analysis

The data reported in the Results section - the mean ± standard deviation (SD) - were statistically evaluated using OriginPro 8.0 software (OriginLab Corporation, Northampton, MA, USA). The One-way analysis of variance method (one-way ANOVA, *post hoc* test: Fisher’s LSD) was used to analyse the significance of the data. Significance levels were indicated as follows: x - *p* < 0.05; y - *p* < 0.01; z - *p* < 0.001.

## Electronic supplementary material

Below is the link to the electronic supplementary material.

Supplementary Material 1

## Data Availability

All data generated or analysed during this study are included in this published article (and its Supplementary Information files).
