# Supplementary material for: Comparative study of the anti-tumour effects of the imipridone, ONC201 and its fluorinated analogues on pancreatic cancer cell line
Source: Sci Rep. 2025 May 7;15:15925. doi: 10.1038/s41598-025-00070-x (PMC12059162; doi:10.1038/s41598-025-00070-x)
Supplement: Supplementary file 1 — Supplementary Material 1 [file 41598_2025_70_MOESM1_ESM.docx]

*Supplementary Material*

**Comparative study of the anti-tumour effects of the imipridone, ONC-201 and its fluorinated analogues on pancreatic cancer cell line**

Zsófia Szász ^1, #^ & Angéla Takács ^1, #^, Márton Kalabay ^1^, Péter Bárány ^2^, Tamás Czuczi ^2^, Antal Csámpai ^2^, Eszter Lajkó ^1^, László Kőhidai ^1,*^

^1^ Institute of Genetics, Cell and Immunobiology, Semmelweis University, H-1089 Budapest, Hungary.

^2^ Department of Organic Chemistry, Institute of Chemistry, Eötvös Loránd University, H-1117 Budapest, Hungary.

**
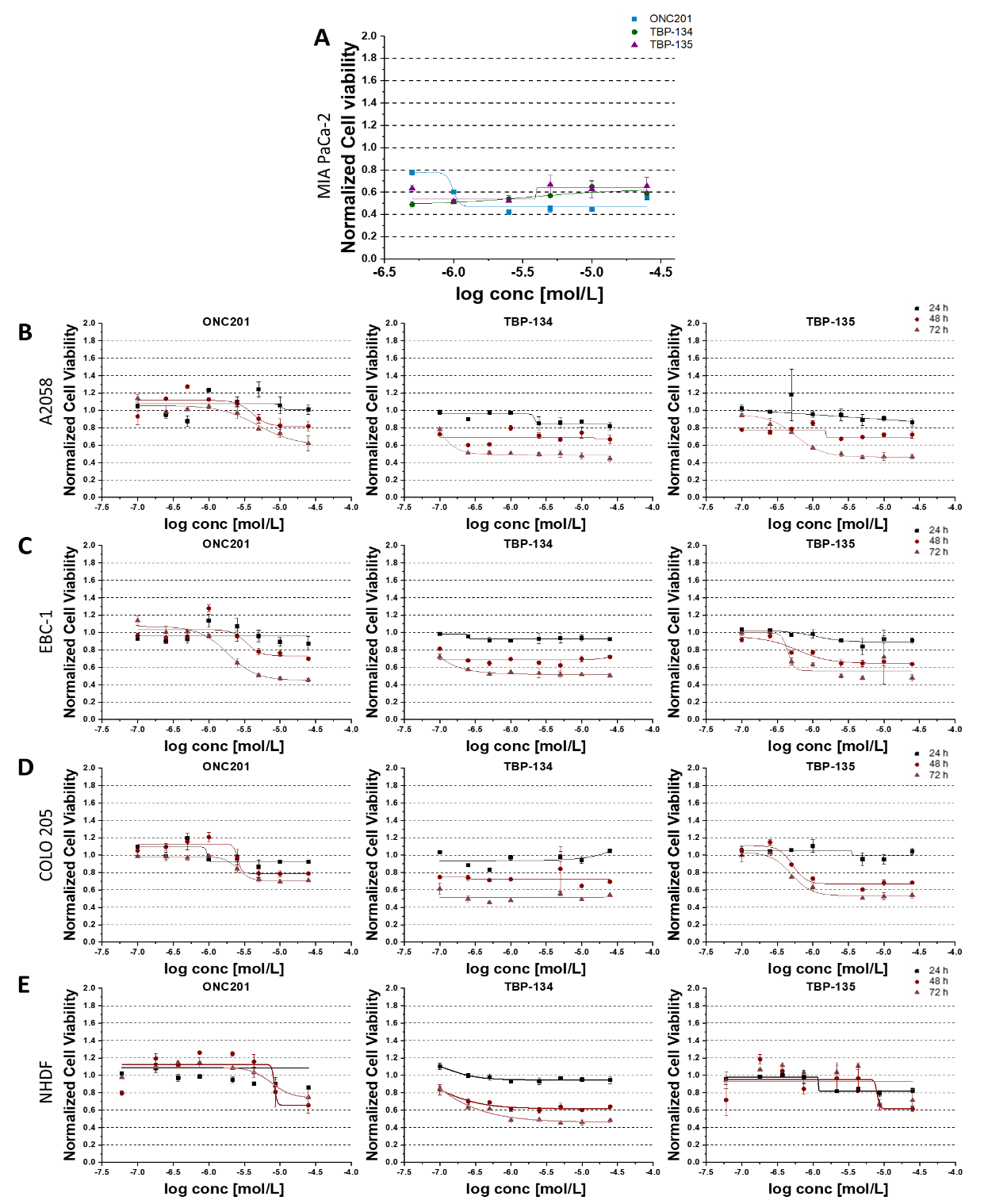
*** Correspondence: [kohlasz2@gmail.com](mailto:kohlasz2@gmail.com)

**Figure S1.** Dose-response curve of ONC201, TBP-134 and TBP-135 72-hour treatment on (A) MIA PaCa-2 cells. Dose-response curve of ONC201, TBP-134 and TBP-135 24-, 48- and 72-hour treatment on (B) A2058, (C) EBC-1, (D) COLO-205 and (E) NHDF cells. The data were normalised to the DMSO control wells The data are presented as mean values ± standard deviation (SD) (n = 3).

**Table S1**. The IC_50_ values of ONC201, TBP-134 and TBP-135 at 72 hours measured on different cell lines.

| **Cell lines** | |  |  |  |
| --- | --- | --- | --- | --- |
|  |  | **IC_50_ values (μM)** | | |
|  |  | **72 hours** | | |
|  | | **ONC201** | **TBP-134** | **TBP-135** |
| **PANC-1** | **human pancreas adenocarcinoma** | **6.10** | **0.35** | **1.8** |
| **MIA PaCa-2** | **human pancreas adenocarcinoma** | **1.12** | **0.67** | **n.d.** |
| **A2058** | **human metastatic melanoma** | **n.d.** | **0.34** | **1.75** |
| **EBC-1** | **human non-squamos lung carcnioma** | **5.72** | **n.d.** | **2.50** |
| **COLO-205** | **human colorectal adenocarcinoma** | **n.d.** | **0.25** | **n.d.** |
| **NHDF** | **normal human dermal fibroblasts** | **n.d.** | **1.55** | **n.d.** |
| **HL-1** | **immortalized mouse cardiomyocyte** | **n.d.** | **n.d.** | **n.d.** |

The abbreviation n.d. stands for not detectable.


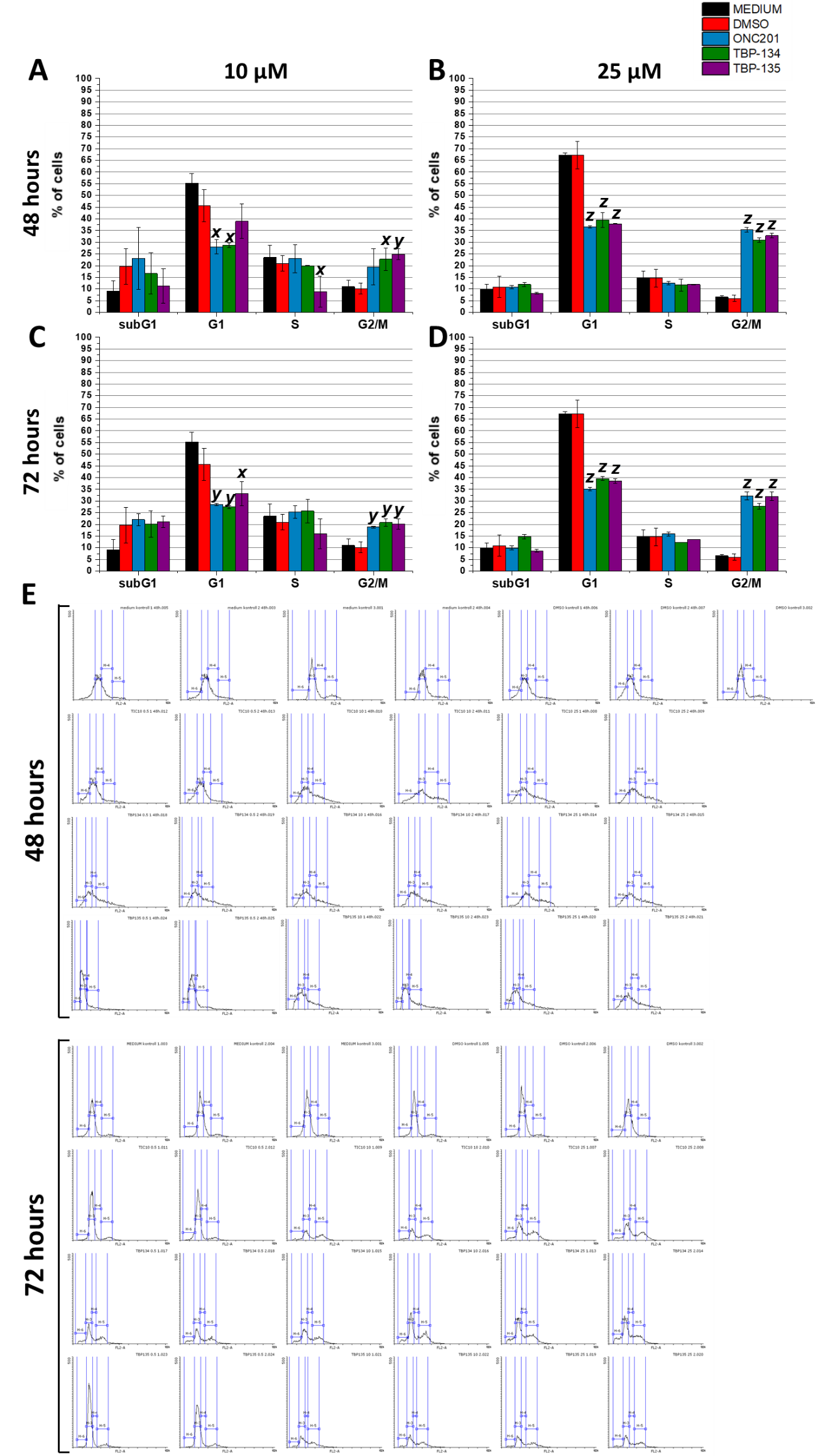


**
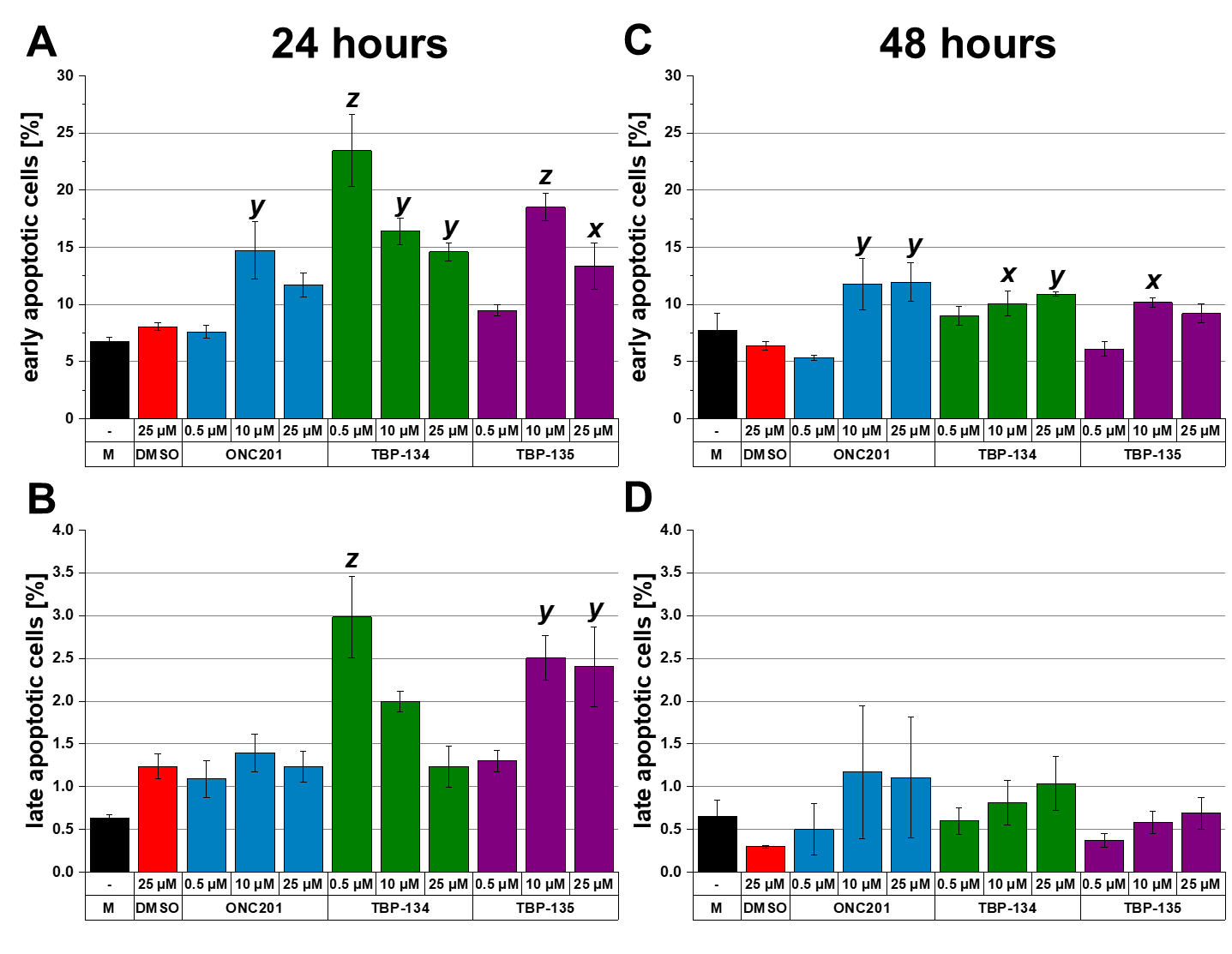

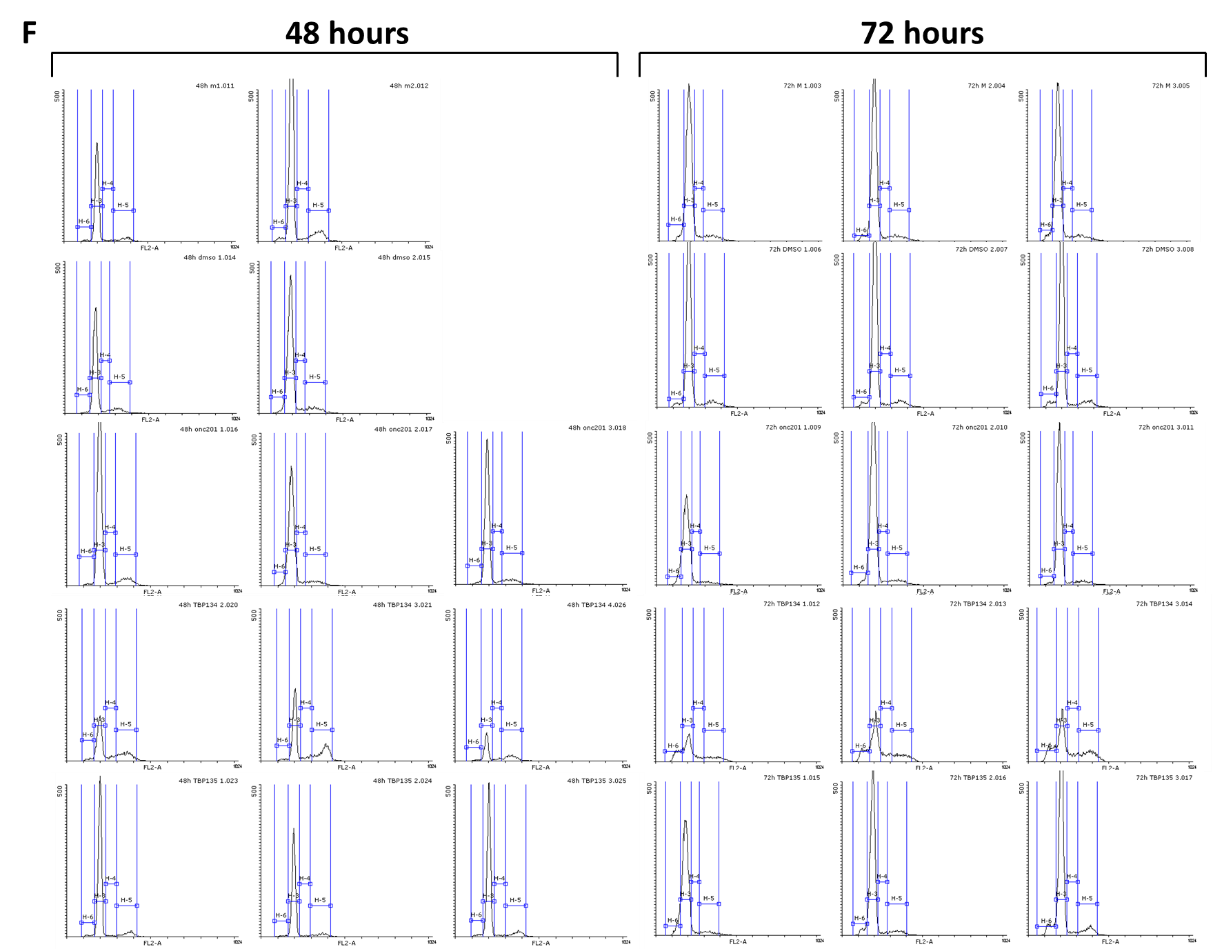
Figure S2.** Cell distribution (percentage of cells) in PANC-1 cell cycle phases shown after (A, B) 48- and (C, D) 72-hour treatment. The data are presented as mean values ± standard deviations (SD) (n=2). Significance levels are calculated to the DMSO control and shown as x - p<0.05; y - p<0.01 using one-way ANOVA test followed by Fisher's LSD *post hoc* test. (E) The histograms showing the FL2-Area after 24, 48 and 72 hours of 0.5, 10 and 25 μM treatment with ONC201, TBP-134 and TBP-135 of PANC-1 cells. (F) The histograms showing the FL2-Area after 48 and 72 hours of 0.5 μM treatment with ONC201, TBP-134 and TBP-135 of MIA PaCa-2 cells.

**
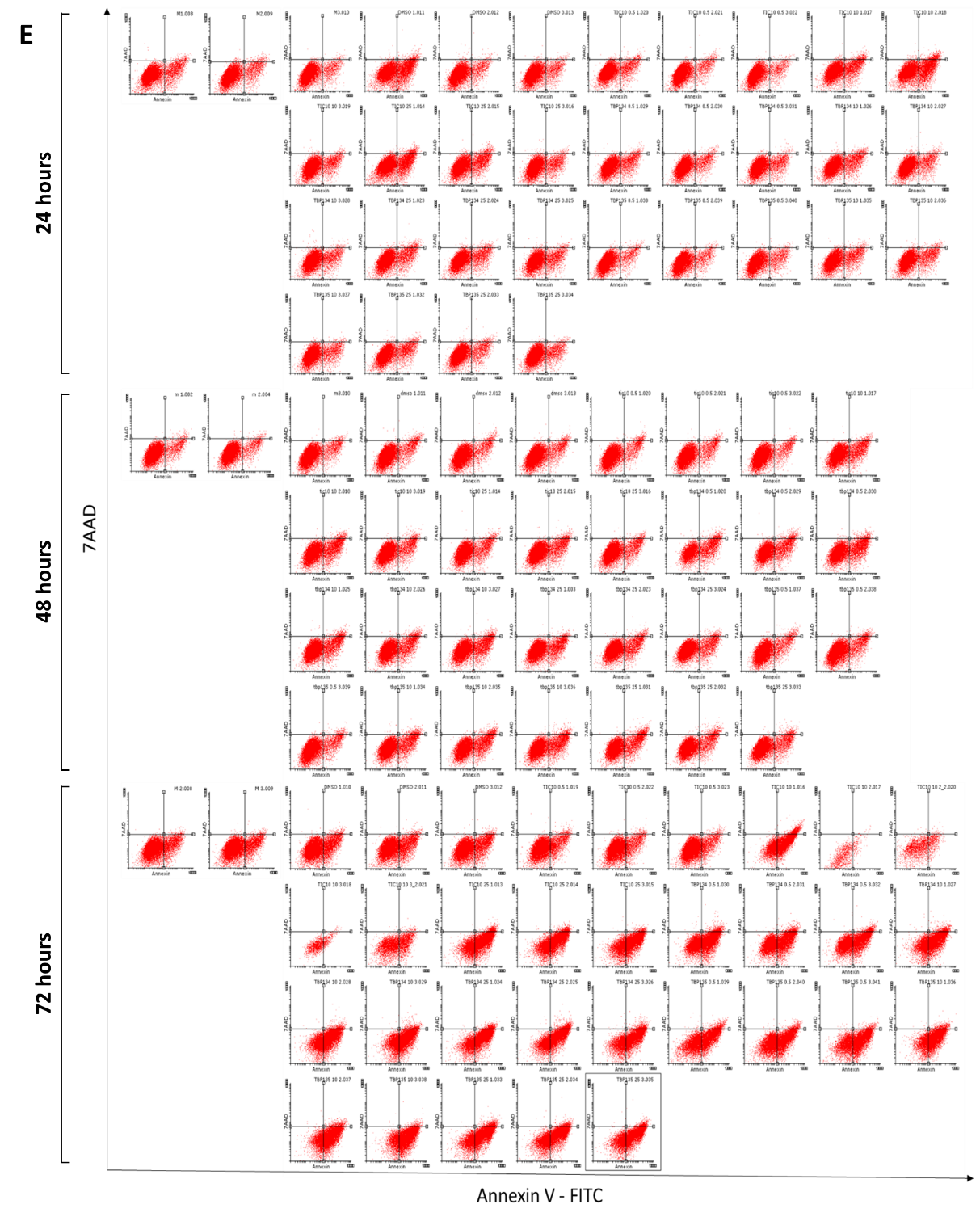
**

**Figure S3.** The apoptotic effects in PANC-1 cells after 24 and 48-hour long treatments with ONC201, TBP-134 and TBP-135. Number of early- and late apoptotic cells normalised to DMSO treatment at (A, B) 24 and (C, D) 48 hours. The data were normalised to the DMSO control wells. The data are presented as mean values ± standard deviaton (SD) (n=3). Significance levels are shown as x - p<0.05; y - p<0.01; z - p<0.001 using one-way ANOVA test followed by Fisher's LSD *post hoc* test. (E) The 7AAD and Annexin V-FITC dot plots after treatment 24, 48 and 72 hours of 0.5, 10 and 25 μM treatment with ONC201, TBP-134 and TBP-135 of PANC-1 cells.

**
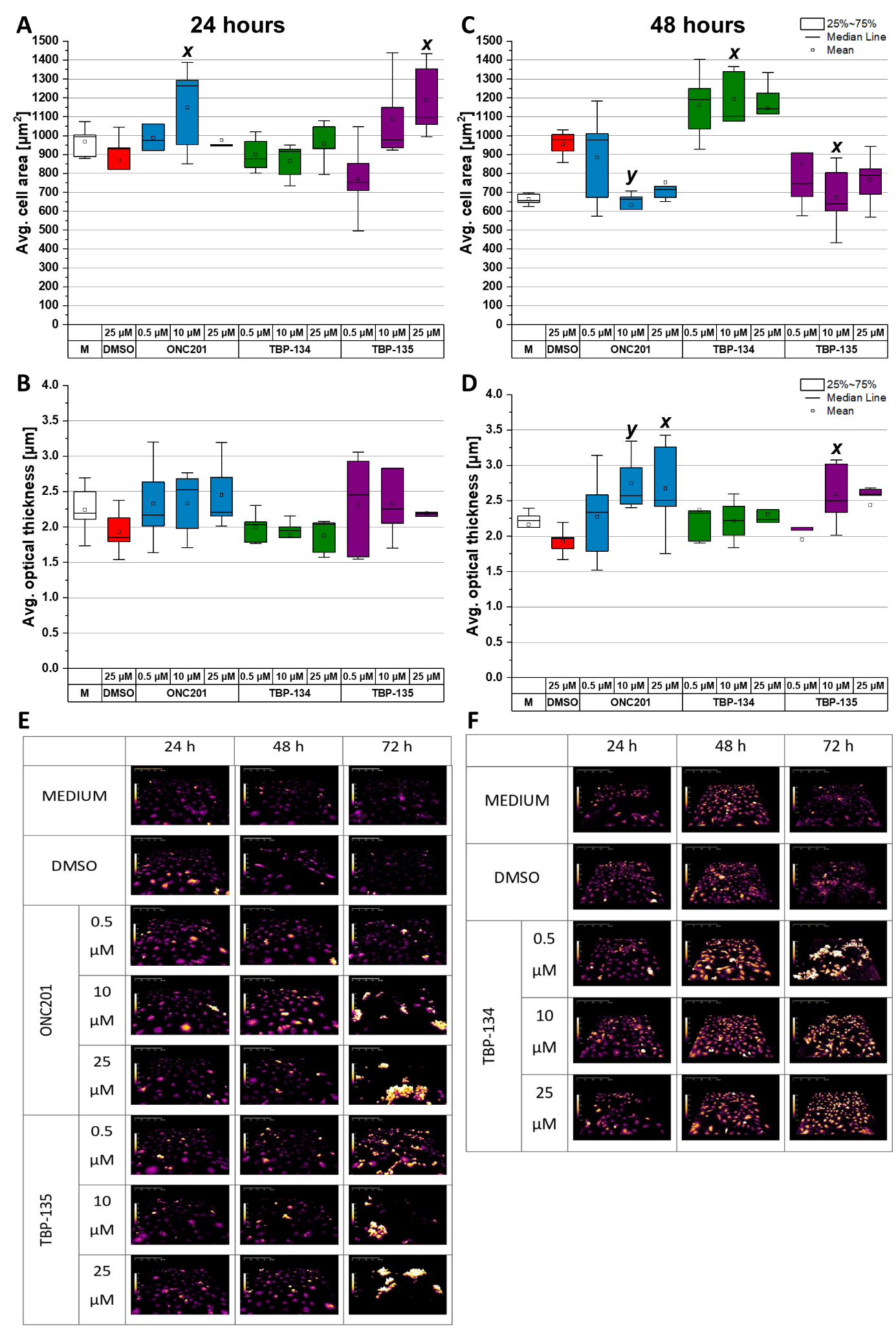
**

**Figure S4.** The morphological changes on PANC-1 cells after 24-, 48- and 72-hour long treatments with ONC201, TBP-134 and TBP-135. (A, C) The average cell area and (B, D) average optical thickness after 24-, and 48-hour treatment shown in box charts. The data were normalised to the DMSO control wells. The data are presented as mean values ± standard deviations (SD) (n=5). Significance levels are shown as x - p<0.05; y - p<0.01; z - p<0.001 using one-way ANOVA test followed by Fisher's LSD *post hoc* test. (E, F) The holographic images taken with HoloMonitor M4 collected in a table.

**
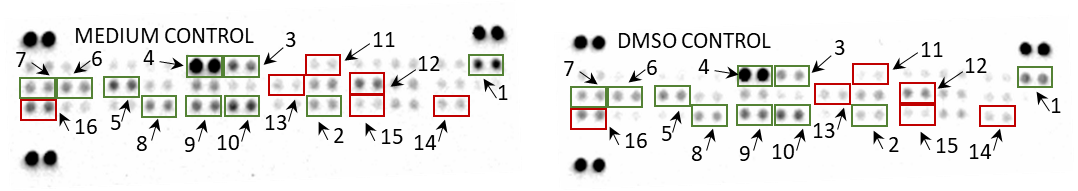
Figure S5.** The proteome profile array blots of PANC-1 after treatment medium and DMSO for the normalisation of the TBP134 treatment. The dots for each protein are duplicates. The green squares indicate the pro-apoptotic proteins, while the red squares show the anti-apoptotic proteins.

**
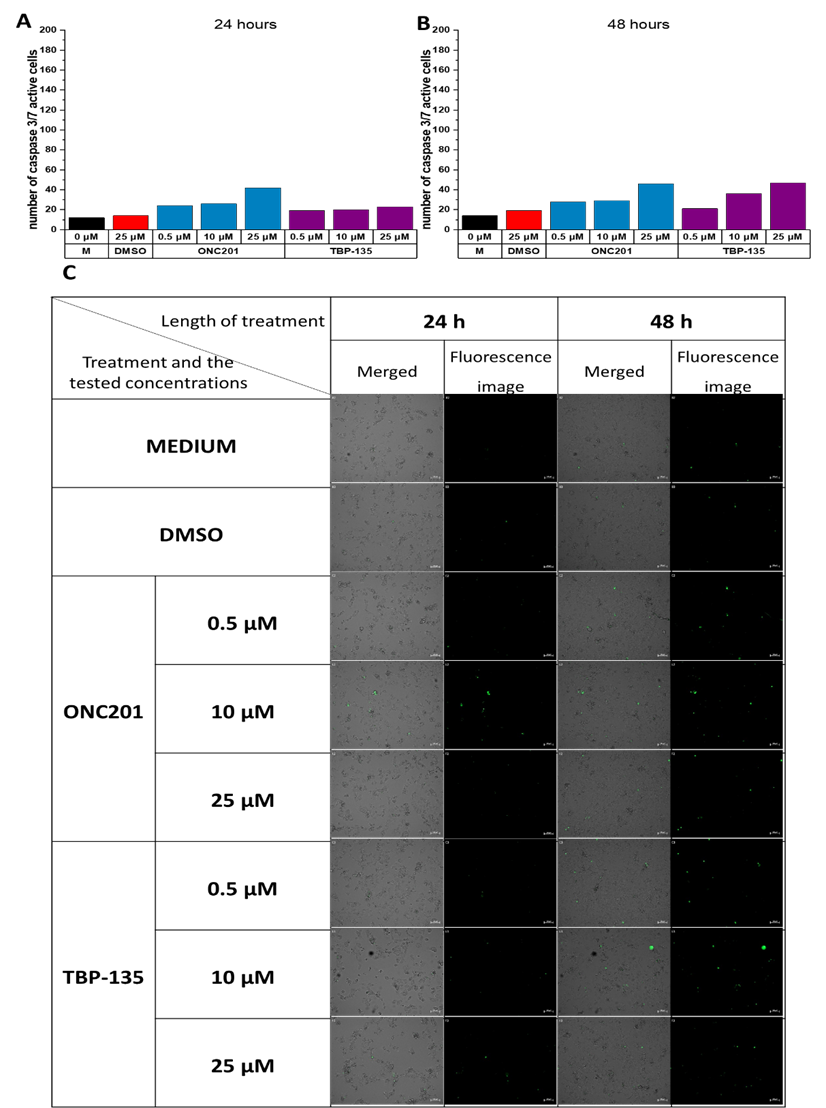
Figure S6.** The number of Caspase-3/7 active PANC-1 cells shown in column charts after. (A) 24 and (B) 48 hours of treatment with ONC201 and TBP-135. (C) The merged brightfield and fluorescence (FITC) channels and the fluorescent channel images of PANC-1 cells taken with Celldiscoverer 7 were collected in a table. The green dots represent the caspase 3/7 active cells.
